# Supplementary material for: Complexome profiling of the Chlamydomonas psb28 mutant reveals TEF5 as an early PSII assembly factor
Source: Plant Cell. 2025 Mar 17;37(6):koaf055. doi: 10.1093/plcell/koaf055 (PMC12164590; doi:10.1093/plcell/koaf055)
Supplement: koaf055_Supplementary_Data [file koaf055_supplementary_data.zip › psb28_interactive.zip!/plots/intensity/qualityPlots/peptides/_Cre17.g697650.t1.1.html]

### Cre17.g697650.t1.1

Trivial name:   
SeqCoverage [median]: 11.10  
Mass: 91.95 kDa  
  
 

| Sequence | MissedCleavage | Mass [Da] | wtIntensity | muIntensity | Score |
| --- | --- | --- | --- | --- | --- |
| DLSTGAELTR | 0 | 1061.54 | 1749.55 | 1228.79 | 81.55 |
| ETDVLDEAEPEEVLLDENARK | 1 | 2413.15 | 0.00 | 949.71 | 30.77 |
| GVDSAAAAAEADR | 0 | 1202.55 | 245.69 | 354.65 | 96.23 |
| IIYISSGSAVTSESR | 0 | 1568.80 | 973.62 | 2667.55 | 113.47 |
| IKEDDTQVPAR | 1 | 1270.65 | 314.55 | 1134.26 | 68.11 |
| VPAAAAAPR | 0 | 822.47 | 439.07 | 0.00 | 68.92 |
|  |  |  | 3722.5 | 6335.0 |  |
